# Analysis of the Physicochemical and Mineralogical Properties of the Materials Used in the Preparation of Recoblocks

**DOI:** 10.3390/ma13163626

**Published:** 2020-08-17

**Authors:** María Neftalí Rojas-Valencia, José Alberto Lopez-López, Denise Yeazul Fernández-Rojas, José Manuel Gómez-Soberón, Mabel Vaca-Mier

**Affiliations:** 1 Institute of Engineering, Coordination of Environmental Engineering, National Autonomous University of Mexico, Av. Universidad, 3000 Mexico City, Mexico; JLopezLo@iingen.unam.mx (J.A.L.-L.); yakult2007@hotmail.com (D.Y.F.-R.); 2 Department of Architecture Technology, Barcelona School of Building Construction, Polytechnic University of Catalonia, Av. Doctor Marañón 44-50, 08028 Barcelona, Spain; josemanuel.gomez@upc.edu; 3 Department of Energy, Metropolitan Autonomous University- Azcapotzalco, 180 San Pablo Ave, 02200 Mexico City, Mexico; mvm@azc.uam.mx

**Keywords:** excavation material, mucilage, construction waste

## Abstract

The construction sector generates 14,000 t/d of construction waste in Mexico City, these materials do not have real applications and end up accumulating in landfills. This work, the objective of which was to analyze the physicochemical and mineralogical properties of soil and construction waste used in the manufacture of Recoblocks, is divided in five stages. First, the excavation material was submitted to field tests. Physical and chemical tests were then carried out on construction waste. Subsequently, the optimal mixture for making Recoblocks was determined. Next, Recoblocks were evaluated and compared with blocks made with water only, without mucilage of *Opuntia ficus,* and finally a feasibility study was performed. The X-ray diffraction study showed the presence of plagioclase, minerals that improve bending resistance, hardness, durability, as well as resistance to stress in a material. Compared to blocks manufactured without mucilage, the use of *Opuntia ficus* mucilage increased the compressive strength of the material by 59%, as well as the erodibility. Recoblocks are an environmentally friendly option because they are based on recycled materials, dried under the sun, which eliminates the use of brick oven. The production cost per unit is just USD 0.19, so it is a viable option as a building material.

## 1. Introduction

For centuries, the construction industry has consumed a significant amount of natural resources; actually, it is one of the main waste generators. It is estimated that more than 10 billion tons of construction and demolition waste are generated in the world [1]. China reported 600 million tons of construction waste in 2017, representing 30% to 40% of the total amount of urban garbage [2]. The European Union estimated that 820 million tons of construction and demolition waste were generated, corresponding to 25–30% of all waste produced in Europe [3,4]. In America, Canada produced more than 9 million tons in 2015 [5]. In total, 548 million tons of construction waste were produced in the U.S.A. at the same year [6]. In Brazil the estimated production of construction waste were more than 70 million tons per year [5]. In Mexico, 6,111,000 tons of construction and demolition waste were generated per year in the period from 2006 to 2012, the latest year for which data are available [7].

Excavation material, concrete and brick are the main components of this type of waste (43%, 24% and 23%, respectively). As observed in various parts of the world, these wastes have a great recycling potential in the place where they are generated, leading to a reduction of the consumption of natural materials [8,9,10].

In recent years, it has been shown that recycled materials are increasingly well accepted by consumers in our countries and throughout the world [11,12].

Properly processed the construction and demolition waste, rise to good quality products, for which a market of great potential has emerged. Local prices, the availability and demand of natural aggregates, as well as the economic situation of the country and the development of the construction industry, are important factors that influence the competitiveness and acceptance of recycled aggregates [10,12,13].

In Brazil, this type of recycling began many years ago; however, its development has been slow, in contrast to the situation in the European Union, where about 70% of construction waste should be recycled by 2020 [9]. In countries such as the Netherlands, Belgium, Switzerland and Austria, this goal has already been reached.

There is a high potential for recycling and material recovery of construction wastes, which so far is under exploited, the level of recycling varies significantly from 10 to 90% [14].

Waste generation in Mexico increased from 30,733,000 tons in 2010, to 42,102,800 in 2012. For 2019, in Mexico City, 14,000 tons of construction and demolition wastes were generated every day [15,16].

Some of the most important uses to reutilize the construction and demolition waste in the construction industry are in hydraulic bases, cycle’s lanes bases and in the foundation fillings which are the places where you need a large volume of material, strategy that is also benefited by the lower prices of the same. The Mexico City Norm [16] indicates multiple uses that have already been tested in different locations in Mexico City [17]. The recycled aggregates easily comply with the standards to be used as road sub-bases [4,18], as construction materials in new buildings and structures or as concrete filler for reconstruction of hydraulic structures [19]. Such use of concrete waste will reduce the volume of extracted natural resources, decrease the load on landfills, and minimize the logistics costs for the transportation of waste and natural resources [8,20,21].

Concretes made with construction and demolition waste have reached compressive strength 30% greater than concrete manufactured with conventional aggregates [22]. Regarding the prefabricated as dividing blocks, curbs and tubes, authors such as [23,24] have studied these products finding results similar to conventional prefabricated with replacement rates between 40% and 60% [25].

As road base and sub-base materials, the results have been very good when mixed with cement to achieve adequate stabilization [26]. The feasibility of using concrete waste for the manufacture of cold asphalt mixtures without altering the mechanical properties has also been evaluated [27], better behaviors have been observed under conditions of high humidity and temperature [28].

On the other hand, concrete waste has been used as raw material for the production of new products such as high quality coarse and fine aggregates [29], cement floor plates [30], coverage for municipal landfills [31], and fly ash for use as additives in high strength concrete [32], and even with the help of heat treatments for cement manufacturing [33]. It has also been possible to improve properties such as fire resistance and thermal and acoustic insulation [25].

There are few reports as regards its composition, so in this investigation complementary studies will be conducted to obtain additional data on this.

To continue promoting the use of construction and demolition waste, this investigation analyzed the physicochemical and mineralogical properties of the materials used in the making of blocks for the construction industry, according to Mexican and European standards.

## 2. Materials and Methods

The procedure followed in this work was divided into five stages. In Stage I, an analysis of the function of the materials used in the elaboration of Recoblocks (blocks manufactured with construction, excavation, logging and mucilage residues) was made. At the same time, a soil mixture (excavation material) from Toluca and Cuernavaca, Mexico was characterized to determine its essential physical aspects. The parameters and procedures used for the physical characterization of the excavation material and construction waste are shown in Table 1.

The characterization of construction waste (6.3 mm to fines, called all in one) was carried out in accordance with Table 2. The procedures followed for analyzing the excavation material were also used for determining waste granulometry and density, while the determination of chlorides, sulfates and total sulfur was performed in accordance with the Spanish standard [39] UNE-EN-1744-1-2013.

In this same stage, the Recoblocks were evaluated and the results were compared to the results obtained from blocks made without mucilage.

The mucilage of cactus is made up of high molecular weight carbohydrates and contains mainly two organic natural polymers: amylase and amylopectin. The amylase is forming a helical chain that in solution has the ability to form films that when drying have high rigidity; while amylopectin, like any high molecular weight compound, has high viscosity but is highly soluble in water. Then, both carbohydrates combined and being in aqueous solution can form layers with different mechanical properties that offer cohesion characteristics and can be used to join materials [40].

The results obtained show that the cactus mucilage improves, in general, the characteristics of the compressed earth blocks due to the reduction of porosity related to a change in the permeability of the solid. The effect of *Opuntia ficus* mucilage on permeability appears to be due to an inhibition in the interaction between water and the solid that does not allow the liquid to rise due to capillarity. The results indicate that the use of *Opuntia ficus* mucilage in Recoblocks increased the compressive strength of the material by 59% compared to blocks that were manufactured without mucilage. The presence of the mucilage also improved the erosion resistance of the blocks in which water penetration is only half of the penetration of the blocks without mucilage. The above can be attributed to the impermeability provided by the presence of the cactus mucilage. These results have been tested in the Engineering Institute laboratory and in other national and international laboratories. Other researchers have found that compressed earth blocks significantly increase dry and wet compressive strength up to 61.5 ± 4.6% [41].

In Stage II, X-ray fluorescence and X-ray diffraction studies were conducted on the excavation material and construction waste.

### 2.1. X-ray Fluorescence Spectrometry

X-ray fluorescence is a technique that allows the identification of chemical elements from the emission of secondary or fluorescent X-rays from a sample that has been exposed to the irradiation of primary X-rays [42].

The different energy states that are unique to each atom allow to determine the elementary composition of a sample with an intensity graph, where each element is identified by a distribution of characteristic energy peaks [43].

For the present work, an analysis of major elements in a molten sample was performed with a sequential X-ray spectrometer (Siemens SRS 3000, USA), using a 10% dry sample concentration. The calcination loss was determined by heating 1 g of the sample at 950 °C for two hours and calculating the mass difference.

### 2.2. X-ray Diffraction of Excavation Material and Construction Waste

X-ray diffraction is a technique that identifies the minerals present in a sample from the X-rays diffracted by the crystals. Since each crystalline structure is unique for each mineral, this implies that diffracted X-rays are also specific for each mineral, permitting to determine the presence of certain minerals in a spectrum [43].

An X-ray diffraction analysis was made to determine the mineralogical composition of the samples using a double-load aluminum sample holder, as well as an EMPYREAN diffractometer (Malvern, United Kingdom) equipped with a Ni filter, fine copper pipe lamp, graphite monochromator and PIXcel3D detector. The measurement was performed in a 2θ angular range from 5° to 80° with a “stepscan” of 0.003° and an integration time of 4 s per step.

The samples were previously crushed and homogenized with a mortar and sifted through a 200 mesh (<75 microns).

Stage III of the project consisted of determining the optimal mixture for making Recoblocks.

After performing field tests to determine the characteristics of the excavation material, and based on previous studies [15,44,45], preliminary material mixtures were proposed in order to observe their behavior and determine the optimal conditions for making Recoblocks. Excavation materials obtained from Toluca, State of Mexico and Tlayacapan, Morelos, with a granulometry of 6.3 mm to fines, were mixed with virgin soil in a 60 to 40 ratio.

Starting from an optimal mixture obtained from the design of experiments, the optimal mixture for the production of Recoblocks was made up of 66% excavation material (soil mixture), 30% construction waste 6.3 mm and 4% felling waste. In addition, water-mucilage material (3:1) equivalent to 20% of the dry weight of the mixture was added for moistening purposes.

To carry out the test, the materials were weighed (with an industrial mixer Tecnoadobe brand, and then dry-mixed for five minutes). For the mixture was added approximately 1.5 L of mucilage, the criterion for this quantity is set until the mixture is moist and moldable by hand, and small Recoblocks were molded, and dried during 48 h. After drying, each block was submitted to compression to determine its resistance.

### 2.3. Description of the Statistical Design of the Experiment

The experimental design for the present investigation was of one factor with two random levels, because its purpose was to evaluate the influence of mucilage on Recoblock compared to blocks made without mucilage.

To determine the number of experimental units, the two proposed levels were considered: Recoblock and mucilage-free block. Based on the applicable regulations, five repetitions were made for each response variable: compressive strength, maximum initial water absorption, compressive strength after exposure in the weathering chamber, and erodibility. Thus, multiplying five repetitions by four response variables, by two levels, a total of 40 experimental units was obtained.

The result of the experiment for each of the variables was analyzed with the software GraphPad Prism 6, performing the unpaired two-tailed T-analysis of the proposed levels for each of the response variables.

The following hypotheses were considered:

**Hypothesis** **0 (H0).** *There is no difference between treatments.*

**Hypothesis** **1 (H1).** *The treatments are different from each other.*

#### 2.3.1. Block Making Procedure

**Figure 1:**
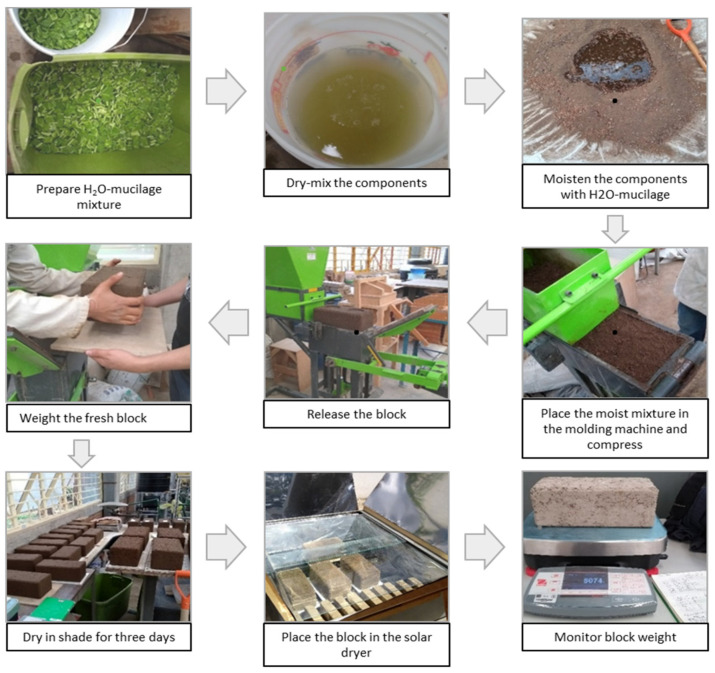
Block making procedure.

The fresh block was weighed and dried in the shade at temperature ambient for 3 days to avoid cracking. Then, it was placed inside the dryer and its weight was monitored, see flow chart in Figure 1.

#### 2.3.2. Equipment Used in the Making of Recoblocks

As mentioned earlier, a block molding machine and a solar dryer are necessary for manufacturing Recoblocks. They are described below:

#### 2.3.3. Block Molding Machine

The Tecnoadobe TA-100 block molding machine is a manual machine for manufacturing compacted earth blocks. According to the technical data sheet, the gross weight of the equipment is 180 kg and its operation requires 3.50 m × 1.50 m of floor space and 2.50 m of ceiling height. It is made of high quality steel and covered with two-tone baked electrostatic paint.

**Figure 2:**
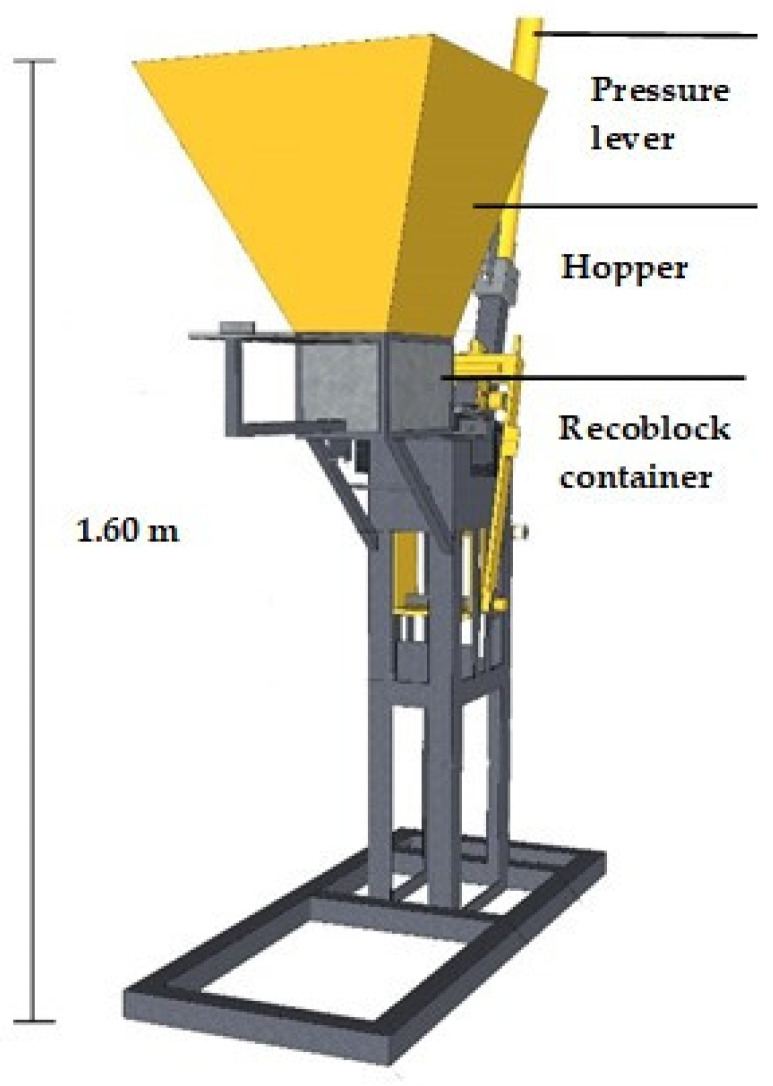
Recoblock molding machine.

Based on the mold, the block is 30 cm long × 15 cm wide × 10 cm high. With this machine, an average productivity of 550 blocks per day can be achieved, according to the manufacturer. Figure 2 shows the block molding machine.

#### 2.3.4. Solar Drier

**Figure 3:**
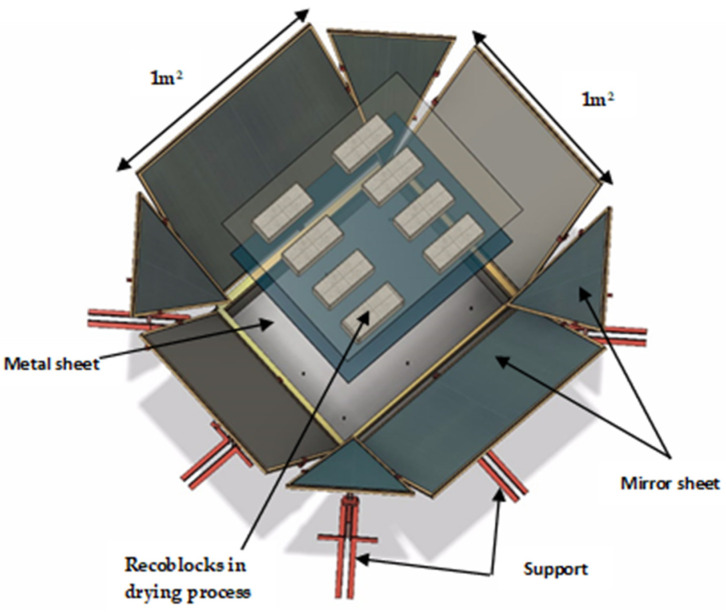
Solar dryer for Recoblocks.

Regarding the solar dryer, it work with a new design which was constructed from recycled elements such as a metal sheet, mirror sheet, support. In order to achieve better sunlight capture, a metal sheet with a moving mirror sheet was used, as can be seen in Figure 3.

### 2.4. Recoblocks Evaluation

After 28 days, Recoblocks were submitted to a series of tests as shown in Table 3.

The first four tests were based on the method described by Aguilar et al., 2017 [44]. The remaining two tests are described in detail below.

### 2.5. Accelerated Weathering Test

**Figure 4:**
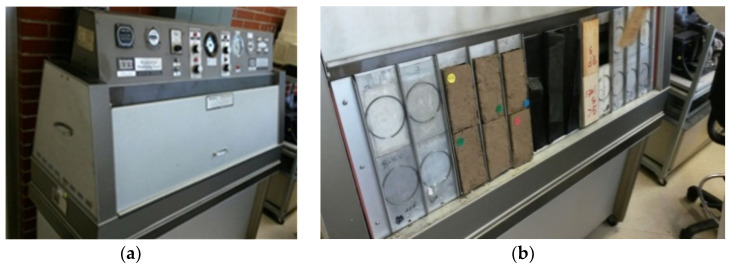
Accelerated weathering chamber Q-LAB, model QUV. (**a**) QUV Chamber with programmable controller, (**b**) Placement of the sample under study, inside the QUV accelerated weathering tester.

With the purpose of simulating weathering conditions, the Recoblocks were introduced in an accelerated weathering chamber Figure 4 (accelerated weathering chamber. Brand: The Q Panel Co. Model QUV (Cleveland, USA). The chamber worked with the following operating conditions: 4 h of exposure to UV light at 60 °C, 4 h of condensation at 50 °C. A UV-B lamp was used and rotated every 400 h.

In the chamber, a 12-min cycle is equivalent to a day under the chosen weather conditions. 840 cycles (168 h) are equivalent to a year under normal weather conditions in Mexico City.

### 2.6. Erodibility

It is well known that land-based building materials tend to lose strength and stability when they are in contact with water for prolonged periods of time [51]. Therefore, in several studies, spray erosion test is used to evaluate the durability of earth blocks [52,53].

On the other hand, pursuant to New Zealand standard NZS-4297-1998 [50], erodibility is established as an additional test for land-based building materials. The test consists of spraying water on one of the faces of a block for an hour or until water penetrates through the specimen. Each 15 min, the test is interrupted to check the depth of erosion caused by water in the block.

The maximum depth is measured one hour after the start of the test. When water bores a hole through the specimen in less than an hour, the rate of erosion is obtained by dividing the thickness of the specimen by the time taken for full penetration to occur. The erodibility index is determined according to Table 4.

### 2.7. Standards Applicable to Brick Making

In Mexico the construction sector is regulated by the “Organismo Nacional de Normalización y Certificación de la Construcción y Edificación (ONNCCE)” (National Organization for Standardization and Certification of Construction and Building). However, it is important to mention that as regards the use of excavation material and construction waste as primary materials in the manufacture of bricks and blocks, no applicable standards were found for manufacturing and quality evaluation. Because of this, the following standard was considered for this investigation.

NMX-C-441-ONNCCE-2013 [53] for non-structural construction parts is a standard that sets the limits of compressive strength and initial maximum water absorption. Table 5 shows compressive strength values. The average compressive strength for construction parts is based on 5 specimens.

Table 6 shows initial maximum water absorption values.

### 2.8. Standards Applicable to Recoblocks Manufacture

Internationally, standards for compressed earth blocks stabilized with lime or cement are available. It is important to mention that only the Spanish standard UNE-41410-2008, cited by Esteve, 2016 [54] “Compressed earth blocks for walls and partitions. Definitions, specifications and test methods” considers the stabilization of the material by natural elements.

Table 7 summarizes the specifications for water absorption and compressive strength established in various international standards.

At international level, Spanish Standard UNE-41410:2008 cited by Esteve, 2016 [54] “Compressed earth blocks for walls and partitions. Definitions, specifications and test methods”, establishes the characteristics that blocks must comply with and the tests to determine them.

This standard defines a compressed earth block (CEB) like a “piece for masonry generally in the form of a rectangular parallelepiped shape, obtained by static or dynamic compression of wet earth, followed by immediate demolding, and which may contain stabilizers or additives to achieve or develop the particular characteristics of the product”.

Finally, in Stage IV, the environmental benefits of the project were analyzed.

### 2.9. Benefits Economic of the Project

An economic analysis of Recoblocks was performed in order to determine the benefits obtained by using recycled materials instead of natural materials.

The initial investment and the unit cost of manufacturing Recoblocks were established in order to demonstrate the economic feasibility of this proposal, based on the current dynamic of artisanal brick manufacturers in Mexico. The estimates were based on the following assumptions: the manufacturer owns the land where blocks are produced, and its manufacturing capacity is 20,000 blocks per month.

## 3. Results and Discussions

In this section, the theoretical results of the fusion of the mixtures are presented, showing the physical and compositional properties of the floors and the construction waste. The mechanical or durability properties are shown and finally the special characterizations such as X-ray diffraction or X-ray fluorescence. Table 8 shows the functions of the various materials used to make Recoblocks.

### 3.1. Physical and Compositional Properties

**Figure 5:**
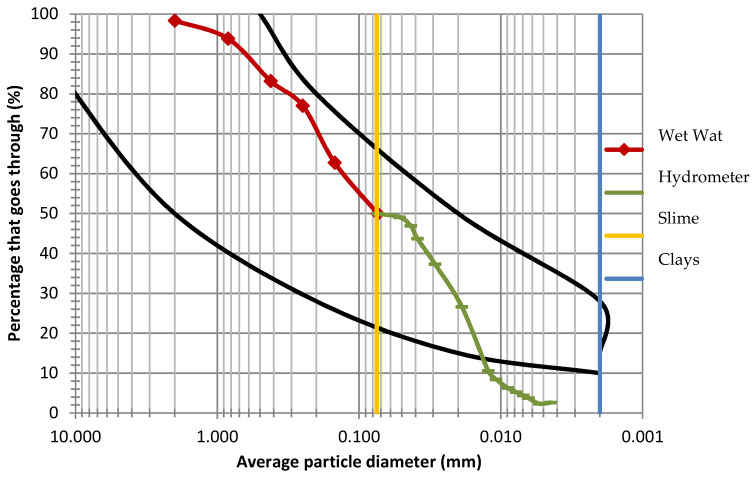
Granulometric curve of the Toluca and Cuernavaca excavation material mixture.

Table 9 shows the results of the characterization of excavation materials and construction waste that were used to make the mixture. With respect to granulometry, it is shown that the major minerals in the materials analyzed are Silts and Sand in the excavation materials, and 93% of sand in the construction materials. The granulometric curve (Figure 5) was constructed by plotting the wet track results, as well as the results of the hydrometer method for fine particles against the opening diameter of the meshes in the case of sieving and the particle diameter for the content of fines.

According to the results, 50% of the material passes through No. 200 mesh and thus, based on the Unified Soil Classification System classification [55], the excavation material can be considered as a fine soil.

On the other hand, in Figure 5, the black curve represents the granulometry limits established in the Spanish standard UNE-41410:2008 [55], where it is recommended that the soil used should contain at least 10% of clay material.

Considering the particle size obtained using the hydrometer method, the excavation material from Toluca could be considered essentially as silt. However, for a more precise classification, it was necessary to determine the Atterberg limits which resulted so the soil mixture could be considered sandy clay of low plasticity.

The organic matter content in the excavation material was 1.076%, which is below the 2% limit indicated in UNE-41410:2008 [55]. The excavation material is thus suitable for making blocks. This result has noticed to be similar with the results obtained by Bisht and Neupane [56], which suggests that organic matter increases with the distance and claims that Organic carbon level greater than 0.8% indicates good quality soil.

The content of soluble salts present in the excavation material was 0.559%, which is below the 2% limit indicated in UNE-41410:2008 [55]. The excavation material is thus suitable for making blocks. In conjunction with data from the literature [17,57,58,59,60] indicated that this material is appropriate to the manufacture bricks.

### 3.2. X-ray Fluorescence Results of Excavation Material and Construction Waste

From the qualitative point of view both samples have the same chemical composition, the major component being SiO_2_ with contents of 64% and 50% for excavation material and construction waste 6.3 mm fines all in one, respectively. The SiO_2_ content allows both materials to be classified as intermediate rocks that are mainly constituted by plagioclase, quartz, feldspars and pyroxene. Table 10 shows the results of the X-ray fluorescence analysis.

The presence of Al_2_O_3_ can be attributed to the ceramics found in both samples, while the greater presence of CaO in construction waste can be attributed to the mortar and cement paste adhered to the bricks [57]. The result of components such as SiO_2_, Fe_2_O_3_ are similar as the ones presented by Kim, 2018 [58]. Talking about CaO and Na_2_O the results obtained by excavation material and construction waste are higher than the ones presented by Kim, 2018 [58].

In addition to the above, the high contents of 2SiO_2_, both in excavation material and construction waste (greater than 50%), fall within the range of 47% to 85% reported in other studies [59,60].

Table 11 shows the results of the X-ray diffraction analysis. These results agree well with the investigations of Bianchini et al 2005 [61], who analyzed different size fractions of recycled fine aggregates by X-ray fluorescence, and determined that there are high contents of calcium compounds in fractions larger than 1 mm.

### 3.3. X-Ray Diffraction of the Excavation Material and Construction Waste

The most abundant minerals correspond to plagioclase that are considered primary aggregates within the tricyclic feldspars of Na and Ca. Actinolite and tridymite were identified in smaller amounts as constituents of intermediate and basic igneous rocks. After feldspars, quartz is the most abundant mineral in the earth’s crust and tridymite is considered a polymorph of quartz (SiO_2_).

The results of the chemical microanalysis show that the proportion of SiO_2_ in all the samples tested is similar to the composition of stone materials that abound in the banks adjacent to Mexico City (dacite and andesite) as reported [62].

As regards the excavation material, it is important to mention that phyllosilicates, minerals associated with clays and relevant for the mechanical behavior of Recoblocks, represent less than 1% of the mixture, that is to say, pure clay represents less than 1% of the excavation material of the mixture. The above corresponds to the result obtained in the granulometric curve of the excavation material which indicates that the fines are silt without the presence of clays.

**Figure 6:**
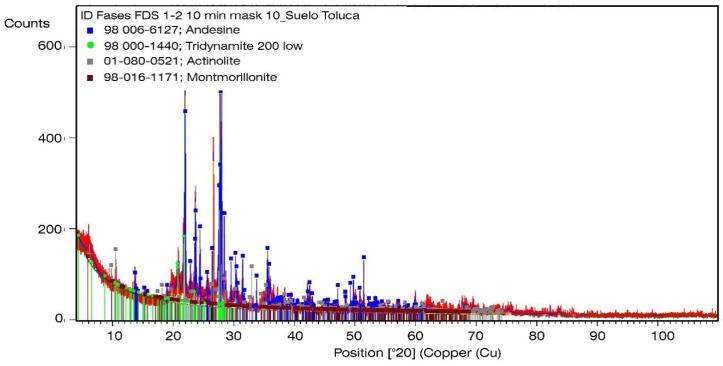
Diffractogram of the excavation material mixture.

**Figure 7:**
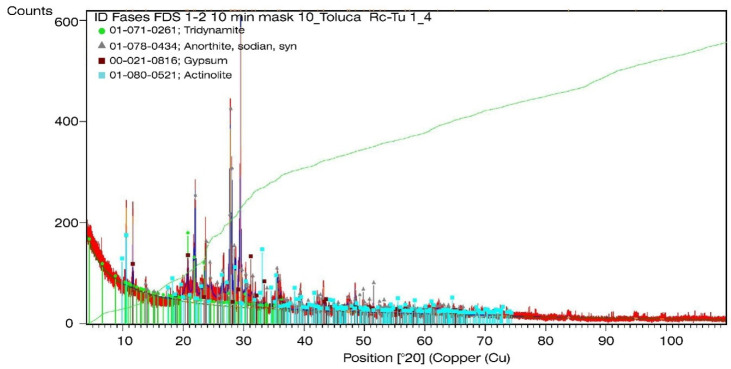
Diffractogram of the construction waste 6.3 mm fines all in one.

Regarding the construction waste 6.3 mm fines all in one, the presence of plaster can be observed in a smaller proportion that can be related to the CaO content reported in the X-ray fluorescence [58]. Figure 6 and Figure 7 show the diffractograms of the excavation material and the construction waste, respectively.

According to the diffractograms, the phases found do not exhibit crystalline behavior because the diffraction peaks are wide with low intensities, which is typical of low crystalline or amorphous phases due to components formed in the hydration of concrete and ceramics that have been crushed [63].

### 3.4. Mechanical or Durability Properties

**Figure 8:**
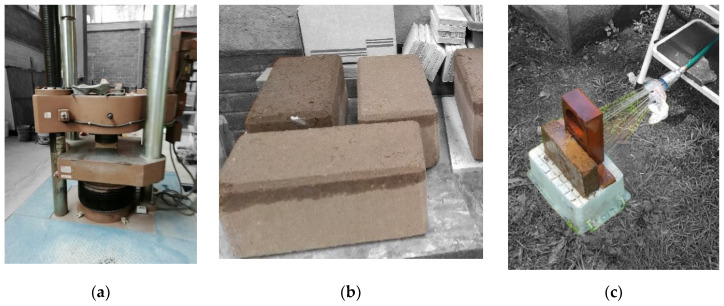
Qualitative results of compressive strength (**a**), initial maximum absorption (**b**) and erodibility (**c**), respectively.

Twenty Recoblocks were prepared. In parallel, other 20 blocks without *Opuntia ficus* mucilage were made for comparison purposes. The results are shown in Table 12, while Figure 8 shows the qualitative results.

Recoblocks showed a 59% greater compressive strength than blocks made without mucilage. Plagioclase improves resistance to bending and impacts, and increase compressive strength, adding hardness, durability and shine to a material. This type of feldspar is used in the ceramic and pottery industry [22].

Moreover, only water blocks showed an initial maximum water absorption 33 (g/min) lower than Recoblocks made with mucilage 21 (g/min). Finally, in the erosion test, Recoblocks showed a greater resistance to water penetration 7 (mm/h), compared to the blocks made without mucilage, which exhibited a water penetration more than twice as high 14.7 (mm/h).

### 3.5. Economic Viability

An analysis was carried out to demonstrate the economic viability of this project. The production cost per unit was obtained taking into account the acquisition price of the equipment, as well as the cost of patent, labor and maintenance of the equipment over 5 years.

Recoblock production cost per unit was USD 0.19, and it is thus a viable building material for interior walls.

## 4. Conclusions

After submitting construction waste to physical, chemical and mineralogical tests, it was determined that the content of sulfates, total sulfur and chlorides present in construction waste was 6.3 mm, which is below the limit established in the Spanish standard UNE-17441.

The use of mucilage *Opuntia ficus* in Recoblocks increased the compressive strength of the material by 59% compared to blocks made without mucilage. The presence of mucilage also improved the erodibility of Recoblocks with a water penetration only half as high as in the case of blocks without mucilage (7 mm/h). The above can be attributed to the impermeability provided by the presence of mucilage *Opuntia ficus*.

Internationally, the resistance of Recoblocks made with a mixture of soils and *Opuntia ficus* mucilage meets the minimum resistance levels established in Brazil, Colombia, Spain, France and Kenya.

The samples analyzed contain different amounts of calcite, which may represent a potential alkaline reserve that serves to stabilize clay soils, so recycled aggregates should not be considered completely inert.

## 5. Suggestions

It is recommended to use the hydrometer method to identify the presence of clay in the soil used.

It is necessary to carry out an evaluation of the adhesion between pieces and mortar, as well as tests on walls built with Recoblocks.

It is advisable to use Recoblocks for interior walls. If they are used for exterior walls, a conventional coating must be applied.

At international level, it is necessary to develop standards that regulate the manufacture of construction materials with recycled aggregates.

A Life Cycle Analysis (LCA) should be performed to show the environmental benefits derived from this construction material.

## Figures and Tables

**Table 1:** Characterization of excavation material.

Physical Characteristics	Procedure
Granulometry Characterization of Fines (should pass through No. 200 mesh)	[34]—Test method for particle size analysis in soils. [34]—Test method for particle size analysis in soils.
Limits of Atterberg	[35]—Test method for liquid limit, plastic limit and plasticity index.
Density	[36]—Test method for specific gravity of soils.
**Chemical Characteristics**	
Content of Organic Matter	[37]—Determination of the content of oxidizable organic matter in a Soil
Content of Soluble Salts of a Soil	[38]—Determination of the content of soluble salts of a soil.

**Table 2:** Characterization of construction waste (6.3 mm).

Physical Characteristics	Procedure
Granulometry	[34]—Test method for particle size analysis in soils.
Density	[36]—Test method for specific gravity of soils.
**Chemical Characteristics**	
Chloride Content Soluble in Water by Volhard’s method	[39]—Tests to determine the chemical properties of aggregates (part 7).
Sulfate Content Soluble in Water	[39]—Tests to determine the chemical properties of aggregates (part 10).
Total Sulfur Content	[39]—Tests to determine the chemical properties of aggregates (part 11).

**Table 3:** Recoblocks evaluation tests.

Performed Tests	Procedure
Determination of Dimensions	[46] Determination of the Dimensions of Blocks
Apparent Dry Density	[47] Determination of net and gross dry density
Compressive Strength	[48] Resistance to compression of blocks
Initial Maximum Water Absorption	[49] Determination of abortion of water
Accelerated Weathering Test	Accelerated weathering chamber
Erodibility	[50] Standards New Zealand

**Table 4:** Erodibility indices.

Parameter	Criteria	Rate of Erosion
Depth of Erosion D (mm/h)	0 ≤ D ≤ 20	1
	20 ≤ D ≤ 50	2
	50 ≤ D ≤ 90	3
	90 ≤ D ≤ 120	4

**Table 5:** Compressive strength values for Mexican standards.

Type of Piece	Configuration	Average Compressive Strength (kg/cm^2^)	Individual Minimum Compressive Strength (kg/cm^2^)
Block	Solid or Hollow	35	28
Extruded Brick	Solid or Hollow	40	32
Artisanal Brick	Solid	30	24
Lattice Piece	Rectangular Face	25	20
	Non-rectangular Face	25	20

**Table 6:** Initial maximum water absorption values for Mexican standards.

Type of Material	Initial Absorption for Exterior Exposed Walls (g/min)	Initial Absorption for Interior Walls (g/min)
Concrete	5	7.5
Extruded or Pressed Clay	5	7.5

**Table 7:** International specifications for compressed Earth block [55].

Country	Initial Absorption	Compressive Strength (kg/cm^2^)
Spain	No water absorption limits indicated	13.3–50.9
Kenya	No water absorption limits indicated	>20.4
Colombia	Cb < 20 g/min, very low capillary absorption Cb < 40 g/min, low capillary absorption	10.2–50.9
France	Cb < 20 g/min, very low capillary absorption Cb < 40 g/min, low capillary absorption	10.2–50.9
Brazil	No water absorption limits indicated	>20.4

**Table 8:** Functions of materials.

Materials	Functions
(a) Excavation Material	Binding material
(b) Construction Waste	Filler material
(c) Wood Waste	Prevention of crack formation during drying
(d) Mucilage *Opuntia ficus*	Formation of layers that increase material cohesion and permeability

**Table 9:** Characteristics of the excavation material and construction waste.

Physical Characteristics	Excavation Material (Soil Mixture)	Construction Waste (6.3 mm Fines)
Granulometry	Sand = 41%, Silts = 54%, Clays = 5%	Sand = 93%, Fines = 7%
Characteristics of Fines	50% of material passes through No. 200 mesh	NA
Limits of Atterberg	LL = 34.9, IP = 11.4	NA
Density	2.74	2.66
**Chemical Characteristics**		
Organic Matter Content	1.076%	NA
Content of soluble salts in soil Water soluble chloride content [54]	0.559%	0.0010% < 0.01%
Water soluble sulfate content [54]	NA	0.0351% < 1%
Total Sulfur Content [54]	NA	0.3285% < 1%

NA = Not applicable, LL = Liquid limit, IP = Plastic limit.

**Table 10:** X-ray fluorescence of excavation material and construction waste.

Component	Unit	Soil Mixture	RC 6.3 mm
SiO_2_	mass %	64.463	50.568
TiO_2_		0.808	0.733
Al_2_O_3_		16.290	13.950
Fe_2_O_3_		4.880	4.635
MnO		0.051	0.077
MgO		1.106	2.580
CaO		3.968	12.503
Na_2_O		3.384	2.307
K_2_O		1.458	1.311
P_2_O_5_		0.126	0.225
PXC		3.460	11.110
Total		99.994	99.999

**Table 11:** X-ray diffraction analysis of excavation material and construction waste.

Phases	Unit	Toluca Soil	6.3 (mm)
Plagioclase (Na, Ca) (Si, Al)_3_O_8_	Mass %	80	68
Actinolite		11	16
Tridymite: SiO_2_		9	10
Phyllosilicates		Traces	-
Plaster: CaSO_4_ 2H_2_O		-	6
Total		100	100

**Table 12:** Results of block evaluation.

Test Performed	Recoblocks	H_2_O-Only Blocks
Compressive Strength (kg/cm^2^)	25.0	15.7
Initial Maximum Water Absorption (g/min)	21	33
Erodibility (mm/h)	7.0	14.7
